# The Role of p53 Signaling in Colorectal Cancer

**DOI:** 10.3390/cancers13092125

**Published:** 2021-04-28

**Authors:** Magdalena C. Liebl, Thomas G. Hofmann

**Affiliations:** Institute of Toxicology, University Medical Center Mainz, Johannes Gutenberg University, 55131 Mainz, Germany; thomas.hofmann@uni-mainz.de

**Keywords:** p53 signaling, p53 pathway, wild type p53, mutant p53, gain-of-function, colorectal cancer, cancer therapy

## Abstract

**Simple Summary:**

The transcription factor p53 is a crucial tumor suppressor that regulates diverse cellular responses to protect against cancer development. Deactivating p53 signaling either by altering p53 regulators or by p53 mutations occurs frequently in human colorectal carcinoma (CRC). Forty-three percent of CRCs harbor p53 mutations that reduce wild-type p53 tumor suppressor activity and often provide neo-morphic functions, which contribute to tumorigenesis. In this review, we summarize wild-type p53 signaling, how it can be deregulated in CRC, and the functional and phenotypical effects of p53 mutations. We also discuss current therapeutic strategies of targeting p53.

**Abstract:**

The transcription factor p53 functions as a critical tumor suppressor by orchestrating a plethora of cellular responses such as DNA repair, cell cycle arrest, cellular senescence, cell death, cell differentiation, and metabolism. In unstressed cells, p53 levels are kept low due to its polyubiquitination by the E3 ubiquitin ligase MDM2. In response to various stress signals, including DNA damage and aberrant growth signals, the interaction between p53 and MDM2 is blocked and p53 becomes stabilized, allowing p53 to regulate a diverse set of cellular responses mainly through the transactivation of its target genes. The outcome of p53 activation is controlled by its dynamics, its interactions with other proteins, and post-translational modifications. Due to its involvement in several tumor-suppressing pathways, p53 function is frequently impaired in human cancers. In colorectal cancer (CRC), the TP53 gene is mutated in 43% of tumors, and the remaining tumors often have compromised p53 functioning because of alterations in the genes encoding proteins involved in p53 regulation, such as ATM (13%) or DNA-PKcs (11%). TP53 mutations in CRC are usually missense mutations that impair wild-type p53 function (loss-of-function) and that even might provide neo-morphic (gain-of-function) activities such as promoting cancer cell stemness, cell proliferation, invasion, and metastasis, thereby promoting cancer progression. Although the first compounds targeting p53 are in clinical trials, a better understanding of wild-type and mutant p53 functions will likely pave the way for novel CRC therapies.

## 1. Introduction

Colorectal cancer (CRC) comprises two common tumor types, namely, colon cancer and rectal cancer, which make up 71% and 29% of CRC cases, respectively [1]. Colon cancer is the fourth most diagnosed cancer worldwide, whereas rectal cancer is the eighth most common type of cancer. Together, these two carcinomas represent the third most common type of cancer globally and the second leading cause of cancer-related deaths. According to estimates of the International Agency for Research on Cancer (IARC), 1.8 million people were diagnosed with CRC and 862,000 people died of CRC in 2018, representing 10.2% of cancer diagnoses and 9.2% of cancer-related deaths, respectively [2].

The major risk factor for developing CRC is age: most CRCs are diagnosed in patients older than 50. In addition to this inherent risk factor, there are other main risk factors, which are lifestyle dependent. These bio-behavioral risk factors include obesity, a sedentary lifestyle, high intakes of red and processed meat, alcohol consumption, and smoking [3]. These risk factors are associated with socioeconomic development and this is reflected in the global incidence and mortality rates, with the majority of CRC cases occurring in countries with high or very high human development indexes. However, the incidence and mortality rates of CRC are rising rapidly in many low and middle-income countries due to lifestyle changes [4]. Additionally, in some high-income countries with decreasing or stable CRCs rates, the burden of early-onset CRC (diagnosis before 50) is alarmingly increasing [5,6]. Thus, CRC represents and will continue to represent a major global health burden.

While 90–95% of CRCs are diagnosed as colorectal adenocarcinoma (COAD), the remaining CRCs represent other subclasses of CRC, including squamous/adenosquamous carcinoma, signet ring cell carcinoma, medullary carcinoma, and endocrine neoplasms [7,8]. The majority of CRC cases occur sporadically (70%), whereas familial or hereditary CRC make up 25% and 5–10% of CRC cases, respectively [9].

One gene frequently mutated in CRC is the tumor suppressor gene p53, which is also known as the guardian of the genome. p53 is activated upon various stress signals, including DNA damage or oncogene activation, and orchestrates a plethora of downstream responses, such as DNA repair, cell cycle arrest, senescence, metabolism, and cell death. p53 mainly acts as a transcription factor controlling the expression of hundreds of target genes [10]. p53 mutations in cancer not only abrogate the ability of mutant p53 to transactivate canonical p53 target genes but may also confer new oncogenic properties contributing to tumorigenesis [11]. Thus, p53 mutant proteins may be considered as oncogenes that actively promote carcinogenesis, although the degree of neo-morphic activity depends on the specific p53 mutation. The observed gain-of-function (GOF) activity might be an explanation for the selection of p53 missense mutations during carcinogenesis rather than nonsense mutations, which are often observed for other tumor suppressor genes.

In this review, we give an overview of p53 signaling, how it is activated in response to stress signals, and which mechanisms regulate p53-mediated cell fate decisions. We discuss how the acquisition of p53 mutations and the deregulation of p53 upstream regulators disrupt p53 signaling in CRC, and how mutant p53 could be exploited as a potential therapeutic target in CRC therapy.

## 2. The p53 Pathway

The transcription factor p53 functions as a central hub that translates various stress signals into diverse cellular outcomes, such as DNA repair, cell cycle arrest, or cell death [12]. The p53 gene is encoded by the *TP53* gene locus located on the short arm of human chromosome 17 (17p13.1). Due to the use of alternative promoters, alternative splicing, and alternative translation sites, at least twelve different isoforms are expressed from the *TP53* locus in a tissue-specific manner. The longest isoform encodes the so-called full-length p53 protein, which is 393 amino acids long and is the best-characterized isoform. Although p53-mediated responses are the endpoints of the activities of the p53 isoforms in specific cell types/tissues, the modes of action of the individual isoforms are only emerging [13,14]. Thus, the contributions of the individual isoforms to a specific p53-response in specific tissues such as the colon and rectum are still unclear.

Full-length p53 possesses seven main functional domains: two transactivation domains (TAD1 and TAD2, aa 20–40 and aa 40–60) at the N-terminus, a proline-rich domain (PRD, aa 60–90), a central DNA binding domain (DBD, aa 100–300), an oligomerization domain (OD, aa 325–356), and a regulatory C-terminal domain (CTD, aa 363–393) [15]. The binding of p53 to DNA is mediated by the DBD, which allows binding to specific DNA target sites termed p53 response elements (REs), and the CTD, which supports sequence-specific DNA binding, especially when the p53 binding site deviates from the consensus p53 RE sequence [16]. The OD is required for the tetramer formation of p53, which is a prerequisite for its activity as a transcriptional activator [17].

### 2.1. Control of p53 Levels

**Figure 1:**
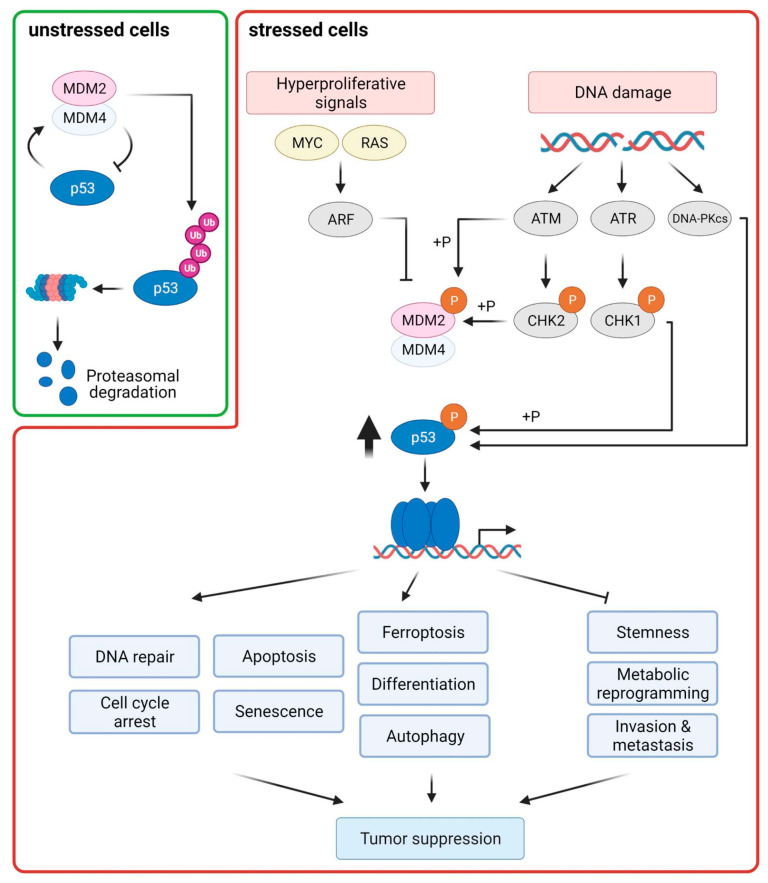
p53 is activated in response to DNA damage and oncogenic stress and coordinates diverse cellular responses that are important for tumor suppression. The transcription factor p53 transactivates MDM2, which is the main E3 ubiquitin ligase targeting p53 for proteasomal degradation, thereby creating a negative feedback loop. In unstressed cells, MDM2 and its partner protein MDM4 are the main regulators of p53 stability. Upon cellular stress, blocking the interaction of p53–MDM2/MDM4 is the major principle for p53 stabilization. DNA damage leads to the activation of the DNA damage response kinases ATM and ATR and their substrates CHK2 and CHK1. Phosphorylation of p53, MDM2, and MDM4 by these kinases stabilizes p53 by antagonizing the p53–MDM2 interaction. In response to oncogene activation, the ARF protein, which is activated by transcription factors of the E2F family, restricts MDM2 activity, thereby stabilizing p53. Activated p53 can modulate many downstream cellular responses mainly by transactivating target genes whose protein products are involved in these processes. Classical outcomes of p53 activation are cell-cycle inhibition, senescence, DNA repair, and apoptosis. Additionally, p53 can regulate other cellular processes, such as promoting autophagy, cellular differentiation, and ferroptosis; and inhibiting invasion and metastasis, metabolic reprogramming, and stem cell self-renewal. All these p53-regulated responses contribute to tumor suppression.

Due to its central role in mediating cellular responses such as cell cycle arrest or cell death, p53 activity must be tightly regulated to avoid inappropriate cellular outcomes. In healthy, unstressed cells, p53 levels are kept low mainly due to the activity of the RING-finger E3 ubiquitin ligase MDM2. The structurally related protein MDM4 (also known as MDMX) interacts with MDM2, prevents p53 transactivation activity, and enhances MDM2-dependent p53 polyubiquitination [18]. MDM2 is itself a transcriptional target of p53, thereby forming an autoregulatory negative-feedback loop [19]. This loop allows the cell tight control over the duration of p53 activation (Figure 1). Although MDM2 is the key regulator mediating p53 degradation, there are also MDM2-independent pathways mediated by other E3 ligases such as PIRH2, COP1, ARF-BP1, and CHIP, which ensure that p53 levels are low in the absence of p53 activating signals [20,21,22,23].

**Figure 2:**
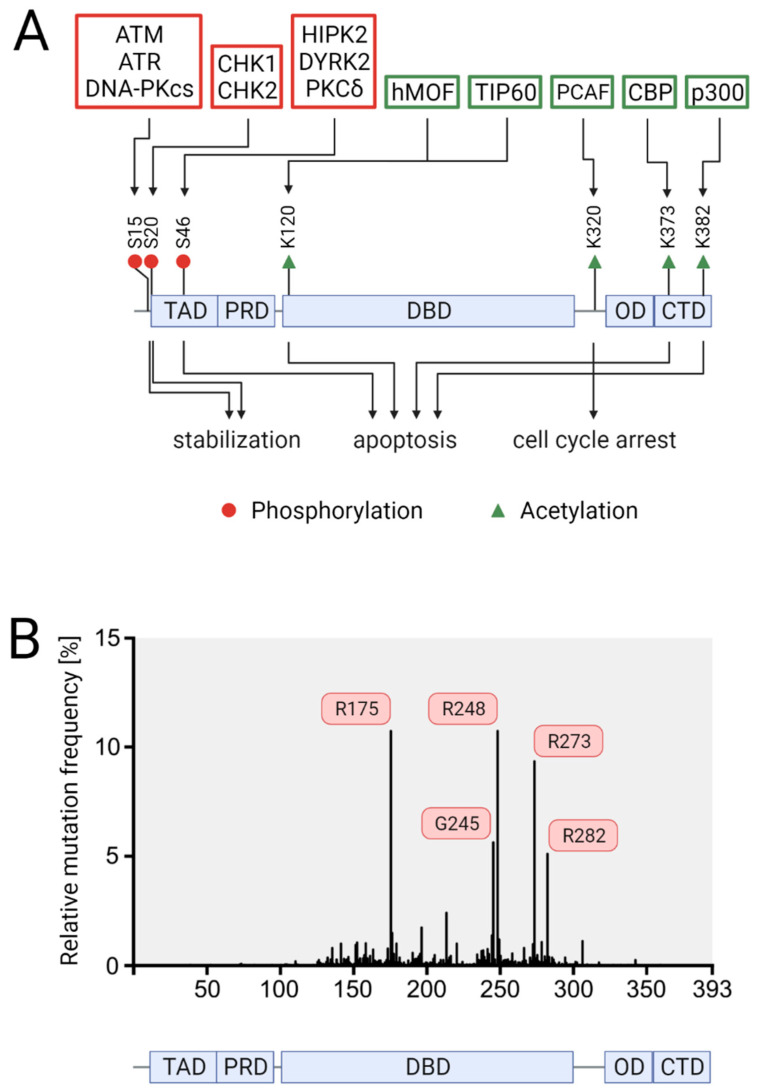
Schematic representation of the domain structure of p53, the impacts of selected post-translational modifications (PTMs), and the prevalence of p53 mutations. (**A**) Effects of selected p53 PTMs on p53 stability and cell fate. The enzymes catalyzing these PTMs are shown above the modified residues, whereas the impacts of these PTMs are depicted below. (**B**) Somatic p53 mutations in CRC according to the IARC TP53 mutation database. A schematic cartoon representing the domain structure of p53. The aligned histogram represents the relative mutation frequency at each position along the p53 protein-coding sequence, based on data of 3607 CRC samples with somatic mutations derived from the IARC TP53 database (R20, July 2019). The five most common mutations are labeled. Transactivation domain (TAD), DNA-binding domain (DBD), oligomerization domain (OD), and carboxyl-terminal domain (CTD).

In response to p53-activating signals such as genotoxic and non-genotoxic stress (e.g., aberrant growth signals), disruption of the p53–MDM2 feedback loop is the main mechanism for rapidly stabilizing p53 [24]. In response to DNA damage, post-translational modifications (PTMs) of both p53 and MDM2 counteract their interaction (Figure 1). The DNA damage checkpoint kinases ATM, ATR, CHK1, CHK2, and DNA-PKcs mediate phosphorylation of p53, MDM2, and MDM4 leading to the dissociation of the p53–MDM2 complex. ATM, ATR, and DNA-PKcs phosphorylate p53 at Ser15, whereas the ATM/ATR substrates CHK1 and CHK2 phosphorylate p53 at Ser20 (Figure 2A) [25]. ATM and DNA-PKcs can additionally phosphorylate MDM2 at Ser395 and Ser17, respectively. MDM4 is a direct substrate of ATM, CHK2, and CHK1; phosphorylation of MDM4 promotes its ubiquitination and degradation [26,27]. In addition to phosphorylation, C-terminal acetylation of p53 counteracts p53 polyubiqutination [28]. Recently, a novel PTM of p53, namely, UFMylation, was reported to antagonize p53 ubiquitination, thereby contributing to p53 stability [29]. In response to oncogenic stress, p53 stabilization is mainly mediated by the tumor suppressor ARF. ARF interferes with MDM2-mediated p53 ubiquitination by constraining the enzymatic activity of MDM2 and/or by sequestering MDM2 in the nucleolus [30].

### 2.2. p53 Downstream Responses

Upon activation, p53 can mediate a diverse set of cellular responses, including DNA repair, cell cycle arrest, senescence, apoptosis, ferroptosis, stem cell reprogramming, invasion and metastasis, autophagy, and metabolism [18,31]. Although transcription-independent functions of p53 are known [32], p53 mainly functions as a transcription factor, which activates the expression of a large and diverse set of target genes. Additionally, p53 can also indirectly suppress gene expression via downstream effectors such as p21, E2F7, and the DREAM complex [33].

How p53 can regulate such heterogeneous outcomes is not completely understood. However, it is known that the cell or tissue type as well as the type and intensity of the stress signal leading to p53 activation are important for the cell fate choice. This is reflected by the fact that out of 3509 potential p53 target genes, which were identified in 17 high-throughput data sets conducted in different cell lines and with different p53 activating agents, only 343 genes were identified in at least three datasets [34]. Mechanistically, it has been shown that post-translational modifications of p53, the interactions with other proteins, which determine the DNA-binding properties of p53, and its temporal expression dynamics are key determinators for p53-mediated cell fate decisions [35,36].

PTMs of p53, such as phosphorylation and acetylation affect promotor-specific DNA binding of p53 and cell fate choice (Figure 2A). In response to severe DNA damage, p53 is phosphorylated at Ser46 by DNA-damage responsive kinases such as HIPK2 and DYRK2 [37,38,39]. This PTM is specifically linked to irreparable DNA damage and leads to the dissociation of the anti-apoptotic protein iASPP from p53, allowing p53 to transactivate a specific set of pro-apoptotic p53 target genes, such as BAX, p53AIP, p53INP1, and NOXA [40,41]. Moreover, phosphorylation of p53 at Ser46 and other residues creates binding sites for the prolyl-peptidyl cis/trans isomerase PIN1 [42,43,44]. Isomerization of p53 at the phospho-Ser46-Pro bond enhances the interaction of p53 with the acetyltransferases CBP and p300 at PML nuclear bodies, which acetylate p53 at Lys373 and Lys382. These PTMs potentiate the transcriptional activity of p53, allowing efficient activation of pro-apoptotic p53 target genes [45]. Additionally, p53, and in particular p53 phosphorylated at Ser46, also exerts non-nuclear, cell death-promoting functions by mediating BAX activation and permeabilization of mitochondria [46]. Another example of a PTM specifying the p53 response is acetylation at Lys120, which is mediated by the acetyltransferases hMOF and TIP60. This PTM enhances the expression of pro-cell death genes such as BAX and PUMA [47,48]. On the contrary, acetylation of p53 at Lys320 by the acetyltransferase PCAF leads to cell survival via activation of the cyclin-dependent kinase inhibitor p21 [49].

The binding of p53 to specific promoters is also regulated by p53-interacting proteins. For example, iASPP and the hematopoietic zinc finger (HFZ) protein stimulate binding of p53 to the p21 promoter, thereby promoting cell cycle arrest, whereas the apoptosis-stimulating of p53 proteins 1 and 2 (ASPP1 and ASPP2) increase the affinity of p53 to pro-apoptotic target genes [50,51,52]. Recently, we identified the adaptor protein DAZAP2 as a new interactor of p53. DAZAP2 inhibits the transactivation of a distinct set of p53 target genes in colorectal cancer cells, including pro-cell death genes upon DNA damage, thereby diminishing p53-induced cell death, presumably in response to repairable genome damage [53].

Another important factor, which determines the outcome of p53 activation, is the temporal change in p53 protein levels, termed p53 dynamics. Rapid induction of p53 levels has been shown to promote apoptosis, whereas slow induction of p53 levels is associated with cell survival [54]. In addition to the induction rate, the dynamics of p53 protein levels are important for cell fate decisions. Cells that display oscillatory behavior of p53 protein levels induce cell cycle arrest and cell survival, whereas cells with a monotonic, sustained increase in p53 levels induce the terminal cell fate choices senescence and apoptosis [55,56]. Pulsatile p53 behavior is mainly generated by the negative feedback loop with MDM2. Indeed, degradation of MDM2 by the caspase-2-PIDDosome has been shown to allow cells to switch from oscillating to sustained p53 dynamics [57]. However, although p53 pulses are associated with pulsatile p53 DNA binding, only the mRNA levels of some target genes show pulsatile behavior, whereas other target genes show a steady increase in mRNA levels. This behavior is determined by the mRNA half-time. mRNAs with short half-times show oscillations in mRNA levels [58,59].

In summary, p53 can induce a heterogeneous response in a cell, tissue, and stress-specific manner, with the option being chosen depending on numerous parameters, such as the presence of interacting and modifying enzymes, and p53 protein level dynamics. These factors regulate p53 target gene expression. Most likely, cell fate choice is not determined by the expression levels of single genes but by the relative expression levels of target gene sets.

## 3. p53 Mutations in Colorectal Cancer

P53 is the most commonly mutated gene in human tumors [60]. Colorectal cancer represents the cancer entity with the highest prevalence of p53 mutations, with 43% of CRCs harboring p53 mutations (IARC TP53 database, R20; https://p53.iarc.fr/TP53SomaticMutations.aspx accessed on 1 April 2021). In contrast to other tumor suppressor genes, which are predominantly altered by truncating mutations, approximately 90% of p53 mutations are missense mutations, meaning that the full-length protein with a single amino acid change is expressed [61]. In CRC, these missense mutations are found at 257 codons in the p53 gene; however, 42% occur at five so-called hotspot codons in the central DNA binding domain, specifically at codons R175, G245, R248, R273, and R282 (Figure 2B). Thus, the distribution of p53 mutations in CRC reflects the one in human tumors in general, which show an additional mutation hotspot, codon R249 [62].

Strikingly, all hotspot codons—except R249—contain CpG dinucleotides. Random deamination of methylated cytosines within those CpG dinucleotides leads to G:C→A:T transitions, which might result in the high prevalence of mutations at these hotspot codons. These G:C→A:T substitutions might also be caused by oxidative stress, which leads to the accumulation of reactive oxygen species (ROS) and subsequent oxidation of DNA bases. The most frequent DNA lesions caused by ROS are 8-oxoguanine and 5-hydroxycytosine, which mispair with adenine and thymine during DNA replication, respectively [63]. High ROS levels have been observed in different diseases, including CRC [64], and might contribute to the prevalence of the p53 hotspot mutations. In line with Knudson’s two-hit hypothesis, which states that both alleles of most tumor suppressor genes need to be inactivated for promoting tumorigenesis, over 91% of tumors show a loss of p53 in both alleles, with the second copy of p53 being inactivated by mutations, chromosomal deletion, or loss-of-heterozygosity [65].

P53 missense mutations are often expressed at higher levels than wild-type p53. How p53 mutants accumulate is not completely understood. However, it has been shown that mutant p53 is impaired in transactivating MDM2 and is additionally more resistant to MDM2-mediated ubiquitination and subsequent proteasomal degradation, allowing accumulation of high levels of stable p53 mutant proteins [66].

### 3.1. Prevalence of p53 Mutations

**Figure 3:**
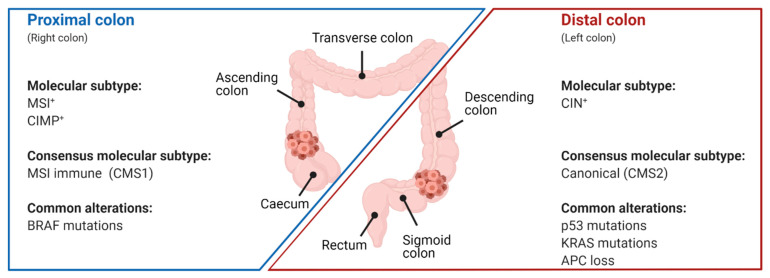
Associations of molecular features with the anatomical location of CRC. Simplified schematic representation of the proximal (caecum, ascending colon, and transverse colon) and distal colon (descending colon; sigmoid colon). Tumors in the proximal colon are associated with microsatellite instability (MSI), CpG island methylator phenotype (CIMP), the consensus molecular subtype (CMS; CMS1), and mutations in BRAF. CRCs of the distal colon are characterized by chromosomal instability (CIN) and CMS2, and often display mutations in p53, KRAS, and APC.

The p53 mutation prevalence rate in CRC varies depending on the age of the patient, the tumor anatomical site, and the tumor molecular subtype (Figure 3). Alterations in p53 are more common in younger (<40 years old) than in older patients [67]. They occur more often in distal colorectal tumors than in proximal tumors, with 45% or 34% of tumors harboring p53 mutations, respectively [68]. This bias of p53 mutations in the distal gut might be due to differences in the microbiome. In a mouse model of CRC, gallic acid prevented mutant p53 from inhibiting pro-oncogenic WNT signaling. Since gallic acid is produced by anaerobic bacteria in the gut (besides the primary production of gallic acid in the liver) and is considerably more abundant in the distal than in the proximal gut, mutant p53 cannot inhibit WNT signaling in the distal gut due to the higher gallic acid concentration, whereas in the proximal gut (with only low concentrations of gallic acid mutant p53) it might even counteract tumorigenesis by inhibiting WNT signaling [69].

The prevalence of p53 mutations also depends on the mutational burden of the tumor. Sixteen to nineteen percent of CRCs are defined as hypermutated based on a high rate of somatic mutations. Two studies analyzed the occurrence of p53 mutations in non-hypermutated versus hypermutated tumors, which were defined as having more than 12 or 17 mutations per one million bases, respectively, and found that 60% of non-hypermutated CRCs harbor p53 mutations, whereas p53 mutations occurred in 20–32% of hypermutated CRCs [70,71]. CRCs are often classified by their genetic and epigenetic aberrations into three subtypes: chromosomal instability (CIN), CpG island methylator phenotype (CIMP), and microsatellite instability (MSI), which are not mutually exclusive [72,73]. p53 mutations are some of the most frequent mutations in CIN-positive CRCs, which usually develop via the so-called canonical pathway following a step-wise progression from early adenoma via late adenoma to adenocarcinoma through acquiring a series of somatic mutations. In this model, p53 mutations are associated with the progression from late adenomas to carcinomas [74,75]. In another subclassification of CRCs based on gene expression by the International CRC Subtyping Consortium, four main consensus molecular subtypes (CMS) were defined. Although p53 mutations were found in all four subtypes, they were most common in CMS2 (62%) and CMS4 (54%). CMS2 comprises the classical type of CRC with high levels of CIN, whereas CMS4 tumors are characterized by a mesenchymal signature [76]. In summary, p53 mutations in CRC are associated with younger patients, distal (left-sided) tumor location, and high levels of CIN.

### 3.2. Functional Effects of p53 Mutations in CRC

**Figure 4:**
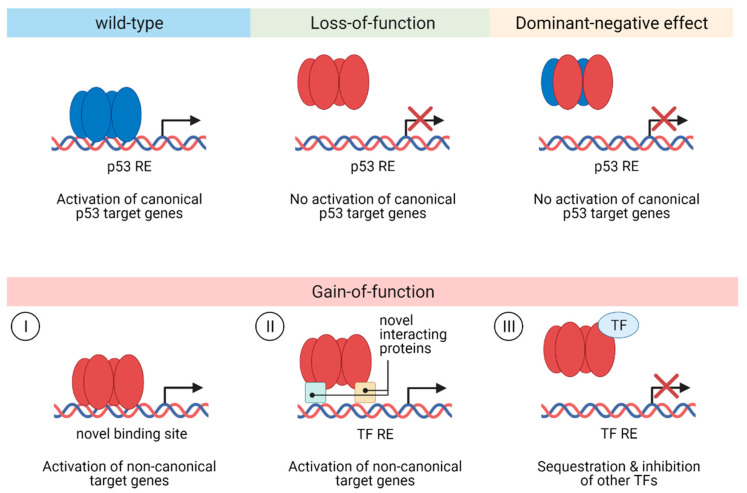
Functional consequences of p53 mutations. p53 mutations can lead to a loss-of-function of wild-type p53 activity abrogating the ability of mutant p53 to transactivate canonical p53 target genes. p53 mutants co-occurring with wild-type p53 can diminish canonical p53 target gene expression via exerting a dominant-negative effect over wild-type p53 by forming hetero-oligomers and preventing binding of wild-type p53 to its response elements. Mutant p53 proteins can also obtain gain-of-function activities by acquiring novel, non-canonical pro-tumorigenic functions through different molecular mechanisms. (**I**) The binding of mutant p53 to novel, non-canonical binding sites leads to the transactivation of non-canonical genes. (**II**) Protein–protein interactions (e.g., with other transcription factors or chromatin remodeling complexes) also induce the expression of non-canonical genes. (**III**) Sequestration of other transcription factors via protein–protein interactions with mutant p53 disrupts target gene expression of those transcription factors. RE, response element; TF, transcription factor.

Missense mutations of p53 usually abrogate all or most of the cellular responses mediated by wild-type p53 (loss-of-function, LOF) and frequently also show a dominant-negative effect (DNE) over wild-type p53 by forming mixed tetramers, thereby impairing the function of a remaining p53 wild-type allele. Additionally, p53 missense mutations can provide the mutant protein with neo-morphic, gain-of-function (GOF) properties, which promote tumor growth and metastasis (Figure 4). While it is clear that p53 mutations lead to LOF, whether DNE or GOF is the key driver behind the spectrum of p53 missense mutations observed in human tumors, including CRC, is still under debate. Over 82% of p53 mutations exhibiting LOF in a saturation mutagenesis screening also displayed DNE, arguing that DNE of mutant p53 over the wild-type protein is important for p53 mutation selection [77]. In another saturation mutagenesis screening, it has been shown that missense mutations, which efficiently inhibit wild-type p53, were highly enriched in a cohort of 1040 patients with myeloid malignancies. Furthermore, in a cohort of 164 patients with acute myeloid leukemia, these missense mutations did not confer an event-free or overall survival advantage compared to patients harboring p53 truncating mutations. Since oncogenic p53 GOF would be expected to give rise to more aggressive disease and worse survival in patients harboring p53 missense mutations compared to patients with p53 truncating mutations, these data suggest that the GOF of mutant p53 is not critical during carcinogenesis [78].

On the other hand, there is also strong evidence for the biological/clinical relevance of GOF properties of p53 mutations. The vast majority of tumors with a p53 mutation show LOH of the second allele, demonstrating that in those cases p53 mutants cannot exert any DNE over an anyway deleted wild-type p53 at least after LOH [65]. An analysis of patients with Li-Fraumeni syndrome, which carry p53 germline mutations, showed that patients with missense p53 mutations had on average a 9-year earlier cancer onset than patients with other types of p53 mutations, such as splicing mutations, non-sense mutations, or frameshift deletions/insertions [79]. However, in another study of Li–Fraumeni syndrome patients, where the authors analyzed the effects of different p53 missense mutations on the age of cancer onset, only the p53 R282 mutation was statistically significantly associated with earlier cancer onset compared to other p53 missense and nonsense mutations [80]. Convincing evidence for GOF of p53 mutations also comes from mouse studies. In a murine tumor model, genetic deletion of mutant p53 slowed cancer growth and extended mice survival [81]. In another mouse study, the authors investigated the GOF properties of two p53 mutants (R172H and R270H, corresponding to human R175H and R273H) expressed from the endogenous p53 locus. p53^mut/−^ mice spontaneously developed a broader spectrum of tumors, including additional carcinomas and more frequent endothelial tumors than p53^−/−^ mice [82], suggesting that mutant p53 manifests GOF biological effects. However, in a mouse model of CRC carrying the p53 R270H mutant, p53^mut/−^ mice showed similar tumor burden, metastasis frequency, and overall survival to p53^−/−^ mice arguing against p53 GOF [83].

The conflicting results concerning DNE and GOF functions of p53 might be due to different cell or tissue types (e.g., myeloid vs. epithelial), different expression levels of p53 (e.g., overexpression of p53 mutant vs. expression from the endogenous locus), lack of intrinsic p53 wild-type expression, or the specific p53 mutation studied. Thus, further studies are warranted to distinguish between DNE and GOF effects of mutant p53.

### 3.3. Gain-of-function of p53 Mutants in CRC

**Figure 5:**
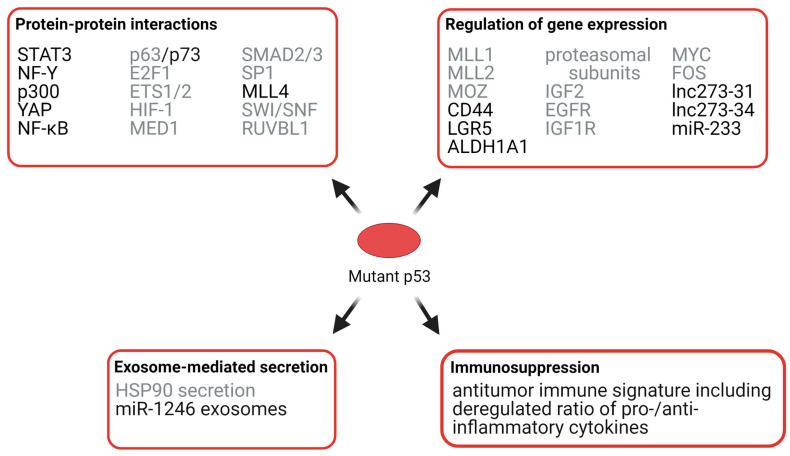
Selected mechanisms of p53 GOF in cancer. Mutant p53 can exert its GOF via protein–protein interactions, the regulation of gene expression, exosome-mediated secretion of biological-active molecules, and immunosuppression. Mechanisms that have been shown in CRC are labeled in black. Mechanisms that have been demonstrated in other types of cancer but might also be involved in CRC are labeled in grey.

Mechanistically, GOF p53 mutant proteins can regulate gene expression via protein–protein interactions with other transcription factors and co-factors, or by binding directly to and transactivating non-canonical, novel target genes. We focus on GOF mechanisms of mutant p53 in CRC, but also describe GOF effects which have been shown in other types of cancer, but to the best of our knowledge have not been investigated in CRC so far and might also be relevant in CRC (Figure 5).

#### 3.3.1. Effects of Mutant p53 on Protein Interactions

Interactions of p53 with other transcription factors can enhance or repress their activity. For example, mutant p53 has been shown to inhibit the transcriptional activity of the p53 family members p63 and p73, resulting in reduced chemosensitivity and enhanced metastatic potential [84]. Mutant p53 proteins can interact with the transcription factor STAT3, which promotes STAT3 phosphorylation, and thus JAK2/STAT3 signaling and proliferation of colorectal cancer cells [85]. By interacting with the transcription factor NF-Y and the co-activator p300, mutant p53 also drives the expression of pro-proliferative target genes in cancer cell lines, including CRC cell lines [86,87]. Together with the transcriptional regulator YAP, mutant p53 can bind to cell cycle target genes in an NF-Y-dependent manner in breast cancer and CRC cell lines, thereby enhancing cell proliferation [88]. Interaction of mutant p53 with NF-Y also induces ephrin-B2 expression, thereby enhancing JNK/c-JUN, SRC/FAK, and SRC/ERK signaling, resulting in enhanced chemoresistance, cancer cell proliferation, and epithelial-to-mesenchymal transition of CRC cells [89]. By augmenting TNFα-induced NF-κB activation in a mouse CRC model, mutant p53 proteins contribute to inflammation-associated CRC development [90]. Additionally, mutant p53 has been shown to interact with various other transcription factors, such as E2F1, ETS1/2, HIF-1, MED1, SMAD2/3, and SP1; these protein–protein interactions have been shown to regulate cell migration, metastasis, angiogenesis, and chemoresistance [84,91,92,93,94,95,96].

#### 3.3.2. Effects of Mutant p53 on Chromatin

p53 GOF mutants can additionally control genome-wide gene expression by regulating chromatin compaction. Mutant p53 has been found to interact with the histone lysine methyltransferase MLL4 in colon cancer cells and stimulate MLL3/4-mediated H3K4 mono-methylation, which is associated with active enhancers [97]. Additionally, mutant p53 can also bind to the SWI/SNF chromatin remodeling complex [98] and RUVBL1, which is associated with several chromatin remodeling complexes, such as TIP60/NuA4, and SWR1-like and INO80 complexes [99]; thus, mutant p53 can control chromatin accessibility and consequently global gene expression.

In addition to interacting with chromatin-modifying enzymes, mutant p53 can also regulate chromatin compaction by transactivating chromatin-modifying enzymes. Mutant p53 has been shown to upregulate the expression of the histone methyltransferases KMT2A (MLL1) and KMT2D (MLL2), which are alternative constituents of the histone H3K4 methyltransferase COMPASS complex, and the histone acetyltransferase KAT6A (MOZ), leading to a global increase in histone methylation and acetylation and cancer cell proliferation [100].

#### 3.3.3. Effects of Mutant p53 on RNA Expression, Exosomes, and Immunosuppression

Although protein–protein interactions of mutant p53 with partner proteins seem to be a major mechanism for the observed GOF of mutant p53, it can also bind directly to specific DNA regions to regulate gene expression of specific genes, such as tumor suppressor genes, oncogenes, and non-coding RNAs. For example, p53 GOF mutants enhance the expression of the colorectal cancer stem cell (CSC) markers CD44, LGR5, and ALDH1A1 by binding to the promoter sequences of these genes, thereby increasing the subpopulations of cells expressing these CSCs markers in colorectal cancer cell lines [101]. Additionally, mutant p53 has been shown to regulate various cellular processes contributing to tumorigenesis by upregulating the expression of genes such as proteasome subunits [102], the growth factor IGF2, the growth factor receptors EGFR and IGF1R, and the proto-oncogenes MYC and FOS [103].

In addition to protein-coding genes, mutant p53 can also regulate the expression of non-coding RNAs (ncRNAs) such as long ncRNAs (lncRNAs, >200 nt length), which can function both as activators or repressors of transcription, and short ncRNAs, such as microRNAs (miRNAs, 18–25 nt length), which act as post-transcriptional repressors of gene expression by binding to complementary regions in the 3’-untranslated region of target mRNAs. These non-coding RNAs can act both in a pro- or anti-tumorigenic manner depending on the cell and tissue types [104]. For example, the lncRNAs lnc273–31 and lnc273–34 are expressed at higher levels in CRCs harboring the hotspot p53 mutations R273H compared to CRCs with wild-type p53 and are associated with cancer stem cell self-renewal, epithelial-to-mesenchymal transition, invasion, and chemoresistance [105]. In breast and colon cancer cell lines, mutant p53 was shown to downregulate the expression of the miRNA miR-233, which contributes to chemoresistance [106].

P53 GOF mutants can also promote tumorigenesis by increasing the secretion of exosomes such as exosome-mediated HSP90α secretion, which enhances tumor invasion and metastasis [107]. Furthermore, p53 mutants can stimulate the secretion of miR-1246-enriched exosomes, which has been shown to contribute to the reprogramming of macrophages to a cancer-promoting state, favoring increased secretion of anti-inflammatory cytokines and possibly contributing to immunosuppression [108]. The association of p53 mutations with immunosuppression in CRC, specifically COAD, has been shown in another study, where the authors demonstrated that tumors with p53 mutations had significantly decreased antitumor immune signatures, including a lower ratio of pro-/anti-inflammatory cytokines than cancers harboring wild-type p53 [109]. In summary, p53 mutations contribute to carcinogenesis by promoting cancer cell stemness, cell proliferation, invasion and metastasis, and immunosuppression. However, it is important to recognize that not all p53 mutants behave equally, but that different p53 mutations may harbor unique GOF activities.

### 3.4. Effects of p53 Mutations on Therapy Response and Patient Survival

p53 mutations are associated with reduced responses to chemotherapeutic agents such as 5-fluorouracil (5-FU), cisplatin, temozolomide, doxorubicin, and gemcitabine; and the anti-EGFR monoclonal antibody cetuximab [110]. In CRC, p53 mutations correlate with chemoresistance to 5-FU, which is a first-line treatment for CRC. One study showed that only patients with wild-type p53 benefitted from 5-FU/levamisole chemotherapy [111]. The association between p53 status and 5-FU resistance has also been observed in vitro and in xenograft mouse models [112,113]. Another study has found that CRC patients with wild-type p53 had better survival when treated with adjuvant chemotherapy than patients carrying p53 mutations [68].

The association between p53 mutations and therapy responses most likely also contributes to the worse prognosis and overall-survival of CRC patients compared to patients with wild-type p53 [71,114]. Progression-free and overall survival was especially poor if the tumors in addition to p53 mutations also harbored KRAS or NRAS mutations, which are frequently mutated in CRC [115,116]. However, other studies did not find a significant correlation between p53 mutation and overall survival in COAD [65,109], which is the most prevalent type of CRC. These discrepancies might be due to different follow-up times of the specific CRC subtype. Taken together, the data linking p53 mutations to survival are currently insufficient; thus, p53 mutations are not considered useful prognostic markers.

## 4. Alterations of p53 Regulators in Colorectal Cancer

**Figure 6:**
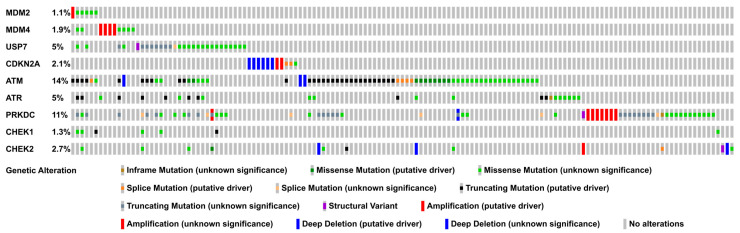
Prevalence of genetic alterations in upstream p53 regulators in COAD. The Oncoprint displays the genomic alterations (legend) in upstream p53 regulators (rows) across 526 COAD samples (columns; TCGA, PanCancer COAD dataset). For the sake of simplicity, only the 143 samples with genetic alterations are displayed. The full Oncoprint including all samples is shown in Supplementary Figure S1. The data were accessed via the cBioPortal webservice (https://www.cbioportal.org/ accessed on 1 April 2021) [131,132].

Tumor cells retaining wild-type p53 can attenuate its function by deregulating genes involved in p53 regulation such as MDM2 and ARF (CDKN2A), or the DNA damage kinases ATM, ATR, DNA-PKcs (PRKDC), CHK1 (CHEK1), and CHK2 (CHEK2). The best-known mechanism to impair p53 signaling in cancer cells is the amplification of its negative regulator MDM2. In many cancers, MDM2 amplification or altered protein expression can be detected [27]. In CRC, one study found MDM2 to be amplified in 9% of cases with MDM2 amplification more often occurring in p53 wild-type tumors [117]. Similarly, MDMX was shown to be overexpressed in approximately 50% of colon cancer tissues [118]. However, when we analyzed the TCGA (The Cancer Genome Atlas) COAD PanCancer Cohort, we found genetic alterations of the MDM2 and MDM4 gene in only 1.1% and 1.9% of samples out of 526 patients (Figure 6; Supplementary Figure S1). These discrepancies might have been due to different cancer subtypes analyzed—CRC, colon cancer, and COAD. Although no subtypes were given for the first two studies, it can be assumed that they were mainly comprised of COAD, as this is the prevalent subtype. Another reason for these conflicting results might have been the method of how the alterations were detected (PCR, Immunohistochemistry, and whole exome/genome sequencing).

When we investigated the mutation prevalence of other p53 regulators, we found that HAUSP (USP7), ATM, ATR, and DNA-PKcs (PRKDC) show frequent genetic alterations in COAD (Figure 6; Supplementary Figure S1). The deubiquitinating enzyme HAUSP is mutated in 5% of COAD samples and has a dual role in p53 regulation, as it can deubiquitinate both p53 and MDM2. Whereas the latter leads to increased p53 ubiquitination and proteasomal degradation, the former has the opposite effect. Since the loss of HAUSP has been shown to lead to an increase in p53 stability, the dominant function of HAUSP seems to be mediated by the ubiquitination of MDM2 [119,120,121]. Thus, it would be important to investigate the effects of the HAUSP mutations observed in COAD on p53 stability.

The DNA damage kinases ATM, ATR, and DNA-PKcs are involved in p53 stabilization and activation and are frequently mutated COAD (14%, 5%, and 11%; Figure 6). These mutations include putative cancer-driving deep deletions, truncating mutations, and missense mutations. Phosphorylation of p53 and MDM2 leads to the disruption of the p53–MDM2 protein complex; thus, this post-translational modification inhibits p53 degradation. As described in Section 2.1, both p53 and MDM2 are substrates of ATM, ATR, and DNA-PKcs. In addition, these kinases can also regulate MDM2 indirectly by mediating the phosphorylation of MDM4. While MDM4 does not have intrinsic E3 ligase activity, it enhances MDM2-mediated p53 polyubiquitination and also reduces p53 transcriptional activity. ATM can directly phosphorylate MDM4 at Ser403. Additionally, ATM, ATR, and DNA-PKcs can indirectly phosphorylate MDM4 via their substrates CHK2, CHK1, and AKT. ATM and CHK2-dependent MDM4 phosphorylation enhances ubiquitination and degradation of MDM4, whereas AKT-dependent MDM4 phosphorylation increases MDM4 stability and thus MDM2 stability [27]. Thus, it would be interesting to analyze whether the ATM, ATR, and DNA-PKcs mutations observed in COAD inhibit the dissociation of the MDM2–p53 protein complex, and thus p53 stabilization and activation.

Cancer cells can also disrupt p53 signaling via the deregulation of non-coding RNAs. Deregulation of ncRNAs can impair p53 signaling by decreasing the levels of p53. Two microRNAs, miR-1827 and miRNA-766, which target MDM2 and MDM4 respectively, were reported to be frequently downregulated in CRC samples implying that their decreased expression in CRC could contribute to impaired p53 stabilization [122,123]. Another miRNA, miR-944, which targets the p53 E3 ubiquitin ligases MDM2 and COP1, was also found to be lower expressed in CRC samples [124]. The long-noncoding RNA PiHL, which is upregulated in CRC, decreases p53 levels by promoting MDM2-dependent p53 polyubiquitination, and thus proteasomal degradation [125]. Two additional ncRNAs were also shown to affect p53 levels: the lncRNA CACNA1G-AS1, which decreases p53 levels, is more highly expressed in CRC tissues than in adjacent normal tissue, whereas the circular RNA, circZNF609, which enhances p53 levels, shows decreased expression in CRC compared with adjacent normal tissue [126,127]. In addition to affecting p53 stability, ncRNAs can also deregulate p53 signaling by inhibiting p53’s ability to transactivate its target genes. miR-214 was found to enhance p53 transcriptional activity. miR-214 shows lower expression in colorectal and cervical cancer tissues than in normal tissue; thus, this miRNA might contribute to dampening p53 transcriptional activity in cancer [128,129]. p53 transcriptional activity can also be regulated by another non-coding RNA, the lncRNA GCln1, which is overexpressed in CRC. GClnc1 reduces the binding of p53 binding to the p21 promoter [130]. In summary, p53 signaling in p53 wild-type CRC cells can be impaired by various mechanisms, including mutations and deregulated expression of upstream regulators.

## 5. p53 as a Therapeutic Target

P53 acts as a double-edged sword in cancer therapy. On the one hand, many chemo- and radiotherapies rely on wild-type p53 activation and its downstream responses to mediate cancer cell growth arrest, senescence, and cell death. On the other hand, mutant p53 has been shown to promote cancer growth and to contribute to therapy resistance [18,62], making both wild-type p53 and mutant p53 interesting targets for cancer therapy. However, for a long time, p53 was considered “undruggable,” mainly due to its lack of a catalytic domain, which could be targeted by low molecular weight inhibitors. This view has changed, and two major strategies of targeting p53 are currently exploited for use in human cancer therapy. In tumors with wild-type p53 (approximately 50% of tumors), activation of p53 to induce p53 outcomes such as cell death in response to cancer therapy is pursued to improve clinical outcomes. In tumors with mutant p53, targeting of mutant p53—for example, by selective degradation or inhibition—is vigorously investigated to counteract the DNE or GOF function of mutant p53 to overcome the cancer growth-promoting effect of mutant p53. In the following sections, we want to introduce the conceptual approaches to target wild-type and mutant p53, focusing on drugs that are in clinical testing. Since a full description of all the strategies targeting p53 is beyond the scope of this article, we recommend the following recent reviews for readers interested in further information [11,133,134,135,136].

### 5.1. Activation of p53 Signaling in p53 Wild-Type Cancer Cells

**Figure 7:**
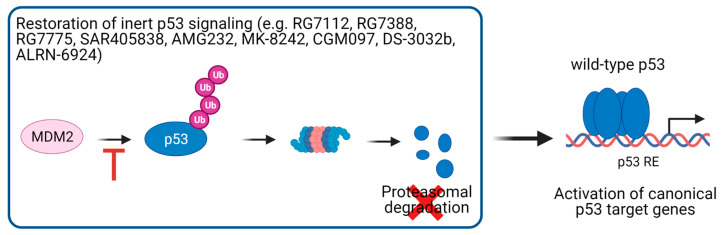
Therapeutic strategies of targeting wild-type p53. In tumors with inert p53 signaling, p53 signaling could be reactivated by using MDM2 inhibitors, thereby rescuing p53 from MDM2-mediated proteasomal degradation. RE, response element.

For cancer cells without p53 mutations, the focus of research was to improve clinical outcomes of cancer therapy by reactivating p53 signaling through inhibition of its proteasomal degradation (Figure 7). Intensive research efforts have led to the development of several molecules inhibiting the interaction of p53 with its E3 ubiquitin ligase MDM2. These antagonists, including the pioneering drug Nutlin-3a, generally bind to the p53 binding interface in MDM2, thereby inhibiting the binding of MDM2 to p53, leading to its stabilization and activation [110,137]. A search of the Clinicaltrials.gov (accessed on 1 April 2021) website showed that the following compounds that block the protein–protein interaction between p53 and MDM2 in CRC or solid cancers, which are not further specified, are currently or have been in phase I/II clinical trials (Table 1): RG7112/RO5045337 [138], RG7388/Idasanutlin [139,140,141,142], RO6839921/RG7775 [143], SAR405838/MI-77031 [144,145], AMG232 [146], MK-8242 [147], CGM097 [148], APG-115 [149,150,151], BI 907828 [152,153], HDM201 [154,155,156,157], and Milademetan/DS-3032b [158]. Additionally, ALRN-6924, which targets both MDM2 and MDM4, is or has been investigated in phase I/II clinical trials in solid neoplasms [159,160,161]. Surprisingly, many clinical studies with MDM2/MDM4 inhibitors do not take the p53 status of the cancer cells into account. This leads to the concern that in patients whose tumors harbor p53 mutations, inhibition of p53 degradation will result in increased levels of mutant p53 proteins, thereby aggravating its DNE and/or GOF effects and posing the danger that these compounds might even contribute to tumor growth and therapy resistance. Thus, stratification of patients according to their p53 status would be advisable when considering the administration of these p53-activating drugs. However, since p53 signaling cannot only be disrupted by p53 mutations, but also by alternative mechanisms, such as MDM2 amplification, the p53 status is not always predictive of sensitivity to MDM2/MDM4 inhibitors. Therefore, p53 gene expression signatures are used as surrogate markers of functional p53 activity [162,163,164,165], which might provide a better assessment of wild-type p53 activity and thus the response to MDM2/MDM4 inhibitors than the p53 mutational status alone. p53 activation might also have unwanted outcomes. In melanoma, it was recently shown that p53 induces slow-cycling cells that contribute to therapy resistance to BRAF/MEK inhibition [166]. Moreover, it will be of particular importance to restrict the p53 activating functions in cancer cells in order to avoid unwanted negative effects on normal, healthy tissues and organs. Thus, specific tumor-targeting of these compounds will be of central importance. Taken together, further research is needed to show the efficacy and safety of p53 reactivating compounds.

### 5.2. Strategies to Target Mutant p53

**Figure 8:**
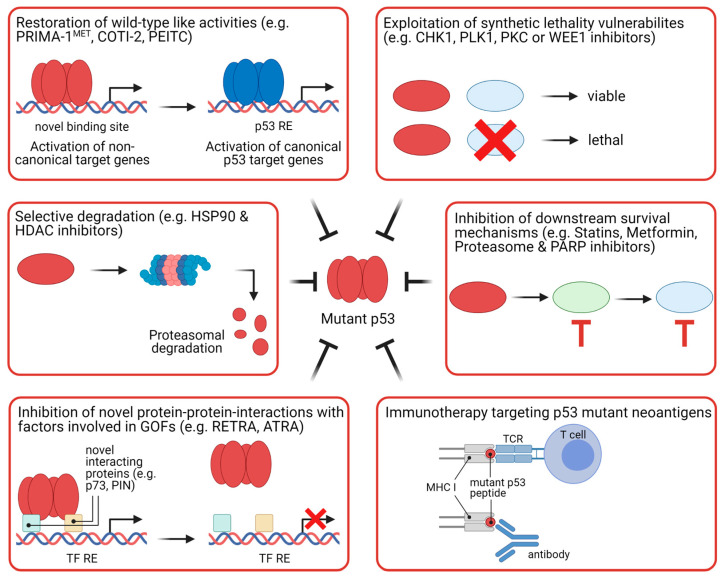
Therapeutic strategies of targeting mutant p53. Approaches targeting p53 mutant that are new or are already in clinical trials can be classified into six categories: restoration of wild-type like activities to mutant p53; selective degradation of mutant p53; inhibition of novel protein–protein interactions involved in mediating gain-of-functions of mutant p53; exploitation of synthetic lethality vulnerabilities of mutant p53; inhibition of downstream survival pathways augmented by mutant p53; and immunotherapy based on the recognition of mutant p53 neoantigens. GOF, gain-of-function; MHC I, major histocompatibility complex class I; RE, response element; TCR, T-cell receptor; TF, transcription factor.

Mutant p53 is a promising therapeutic target since approximately 50% of all tumors harbor p53 mutations, implying that potential therapies could be applied for a wide range of human tumors [167]. Additionally, p53 mutant proteins often accumulate in tumor cells, whereas normal cells only have low amounts of wild-type p53 [66]. This difference in expression raises the hope that drugs against mutant p53 preferentially target cancer cells carrying p53 mutations. Different approaches to target mutant p53 are exploited. The main strategies include restoration of wild-type p53 activities, selective degradation, inhibition of novel protein–protein interactions with factors involved in neo-morphic responses, inhibition of downstream survival mechanisms, exploitation of synthetic lethal vulnerabilities, and immunotherapy (Figure 8).

Restoration of wild-type p53 function aims at inducing the native conformation of the DNA binding domain so that mutant p53 can bind to p53 REs and transactivate canonical p53 target genes to induce cell cycle arrest and cell death. Studies in murine cancer models have shown that wild-type p53 induction leads to tumor regression and prolonged survival [168,169,170], raising the hope that this will be a successful strategy for treating human cancers. Intensive research efforts have led to the discovery of several compounds that can reactivate p53 wild-type activities. Many of these drugs are either cysteine-targeting compounds or Zn^2+^ chelators. To our knowledge, of the various Cys-binding compounds, only PRIMA-1^MET^ (APR-246) has progressed to clinical trials (Table 1). One phase I/II study is currently testing administration of PRIMA-1^MET^ together with the immune checkpoint inhibitor pembrolizumab in patients with solid tumors [171]. Mechanistically, PRIMA-1^MET^ binds covalently to Cys-residues in the core domain of p53 [172]. PRIMA-1^MET^ has been shown to enhance refolding of mutant p53, induce p53 canonical target genes and inhibit tumor growth in murine cancer models [133]. Another approach to induce p53 “wild-type” conformation is by using Zn^2+^-chelating agents. Zn^2+^ is required for wild-type p53 structural stability, and Zn^2+^-chelators have been shown to facilitate the incorporation of zinc into misfolded p53 proteins, thereby restoring the wild-type-like folding and specific DNA binding of mutant p53 [135]. The only drug of this class of compounds in clinical trials is COTI-2 [173]. Another compound that can restore p53 wild-type-like conformation is the food-derived phenethyl isothiocyanate (PEITC), which is currently tested in clinical trials with cancer patients [174].

Induction of mutant p53 degradation, thereby ameliorating its pro-tumorigenic functions, is another strategy for therapeutic targeting of mutant p53. This strategy is supported by the observation that cancer cells can become addicted to mutant p53 [81]. Several compounds have been discovered that can destabilize p53 mutant proteins. These include HSP90 and HDAC inhibitors. HSP90 inactivates the p53 E3 ubiquitin ligases, MDM2 and CHIP, thereby increasing mutant p53 levels; HSP90 chaperone activity is enhanced by HDAC6-mediated deacetylation [175,176]. Thus, targeting of HSP90 or HDAC6 induces mutant p53 degradation. Whereas several HSP90 inhibitors are currently in clinical trials, HDAC inhibitors have already been approved by the FDA for use in cancer therapy [177].

Amelioration of the functions of mutant p53 can also be achieved by blocking protein–protein interactions with factors involved in GOF responses. As described in Section 3.3, mutant p53 can inhibit p63/p73 activity by forming protein complexes with these TFs. The small molecule RETRA can disrupt the mutant p53–p73 complex, leading to p73-dependent transcription and cell death. RETRA has been shown to inhibit tumor growth of mutant p53-expressing cancer cells in a mouse xenograft model [178]. 

In another study, it was demonstrated that the peptidyl-prolyl cis-trans isomerase PIN1 enhances the inhibitory effect of mutant p53 on p63 [179]. Isomerization of mutant p53 also promotes its interaction with the proto-oncogene MYC [180]. PIN1 can be targeted for degradation by all-trans retinoic acid (ATRA), which is approved for the treatment of acute promyelocytic leukemia [181]. However, PIN1 also plays an important role in the activation of the cell death-driving p53 Ser46 kinase HIPK2 [182], which also potentiates cell death induction in the absence of p53 [183,184], suggesting that its use could potentially exert opposite effects.

Inhibition of survival pathways regulated by mutant p53 is another strategy pursued to target cells harboring p53 mutations. One such example is the mevalonate pathway, which is a metabolic pathway providing isoprenoids such as cholesterol and steroid hormones. While wild-type p53 suppresses this pathway, it was found to be upregulated in p53 mutant cells [185,186]. The mevalonate pathway can be inhibited by statins, which are approved for the treatment of cardiovascular disease [187]. Other approved drugs that might be repurposed for targeting mutant p53 are metformin, proteasome inhibitors, and PARP inhibitors, which are approved for the treatment of type 2 diabetes mellitus, multiple myeloma, and BRCA-mutated cancers, respectively [11]. This concept is supported by the observations that mutant p53 drives the Warburg effect, which might be counteracted by Metformin; that mutant p53 induces the transcription of proteasome genes; and that mutant p53 increases PARP1-dependent poly-ADP ribosylation of proteins [102,188,189]. Repurposing already approved drugs for treating cancers harboring p53 mutations is a promising approach since the (sufficient) safety of these drugs has already been proven and drug repositioning might lead to shorter development times due to the reduced number of required preclinical/clinical testing.

Another strategy to target mutant p53 is the induction of synthetic lethality. The rationale behind this approach is that inhibition of pathways on which p53 mutant cells rely for survival leads to a loss of viability. It has been shown that the inhibition of kinases involved in the G2/M checkpoint such as CHK1, polo-like kinase 1 (PLK1), protein kinase C (PKC), and WEE1 induces synthetic lethality in p53 mutant cells [190,191,192,193]. Inhibitors of these kinases are being tested for cancer therapy in clinical trials [11]. The exploitation of synthetic lethal vulnerabilities of cancer cells harboring p53 mutations should result in specificity for mutant p53-expressing cancer cells and spare normal cells expressing wild-type p53. However, since many of the compounds which induce synthetic lethality in cells with p53 mutations are involved in the DNA damage response, this strategy poses the danger of exacerbating genetic instability, which might even drive cancer growth and therapy resistance.

Since p53 mutations do not occur in normal, healthy cells, they might serve as tumor-specific neoantigens. As anti-p53 antibodies have been detected in cancer patients (including patients with CRC) and antibodies and T-cell receptors can recognize p53 mutants, vaccines targeting mutant p53 are being evaluated in clinical trials for the treatment of many different types of cancer, including CRC [194,195,196,197,198]. Unfortunately, clinical studies with p53 vaccines so far have not shown convincing results. This might be due to immunoregulatory and -suppressive mechanisms. Therefore, stimulation of the immune response—for example, by immune checkpoint inhibitors—might improve the efficacy of p53 vaccines [196].

Developing therapeutic approaches against mutant p53 would be of huge clinical benefit due to the prevalence of p53 mutations. However, it is becoming increasingly clear that p53 mutants differ substantially in their function; thus, a “one-size-fits-all” approach will very likely not be applicable.

## 6. Conclusions and Future Perspectives

Decades of research on the function of the p53 protein have shown that p53 is a central hub for integrating diverse cellular stress signals to mediate appropriate cellular outcomes. How p53 controls stress-specific responses remains puzzling. Although the molecular mechanisms controlling cell fate decisions such as post-translational modifications, interactions with cofactors, and p53 expression dynamics are beginning to emerge [36,41,199], more research is needed to understand how p53 translates a specific stress signal into a particular cellular response. Another unsolved question is how p53 is regulated in different human tissues. So far, most of our knowledge about the p53 pathway comes from studies in cell lines and mouse models—two model systems showing limitations. Along these lines, the use of human cancer organoids, which appear to closely match the situation in the tumor, might be of great advantage to understand p53 function in a more cell type-specific manner and to test for therapeutic options [200].

New high-throughput and single-cell technologies will enable the investigation of p53 regulation and responses in a tissue and cell-type-specific manner with only short or no culturing of cells required. This knowledge about context-dependent effects will deepen our understanding of p53 signaling in the colon and rectum, and might be used for manipulating p53 signaling specifically in colorectal cancer cells while avoiding the harmful consequences of targeting p53 in all tissues. 

Another open question is how p53 suppresses tumorigenesis. While previous studies indicated that p53 mediates tumor suppression via induction of cell cycle arrest, apoptosis, and senescence, new research has challenged this view. Instead of a single cellular response, p53 most likely inhibits carcinogenesis by activating numerous cellular responses, including non-apoptotic cell death, migration/invasion, metabolism, DNA repair, and stemness [201,202]. Elucidating the mechanisms by which p53 suppresses tumorigenesis might open the path for new therapeutic options with fewer side effects. Investigating p53-mediated tumor suppression in a cell type-specific manner by the above-mentioned methods and organoid models might also help us understand why p53 mutations are more common in the distal colon than in the right colon.

The crucial role of p53 in suppressing tumorigenesis is highlighted by the fact that 43% of all CRCs harbor p53 mutations. Potentially all CRC patients could benefit from therapies targeting p53. Therapeutic strategies aiming at reactivating inert p53 signaling in tumors with wild-type p53 on the one hand, and restoring wild-type like activities of mutant p53 on the other hand, are in clinical trials and if successful will have a huge clinical impact. Whereas there are several compounds in clinical trials which can reactivate wild-type p53, only two drugs targeting p53 mutant proteins directly are being investigated in clinical trials. This highlights the fact that further research is needed on how to target mutant p53. However, since it is becoming increasingly clear that different p53 mutations vary in their functionality and pathobiology, individual therapies targeting the different classes of p53 mutations might have to be developed. In addition, since many clinical studies with MDM2/MDM4 inhibitors did not take the p53 status of the cancer cells into account, better stratification of patients according to their p53 status is required for testing the efficacy of p53-activating drugs.

Since targeting/activating wild-type p53 is not trivial owing to massive side effects due to the function of p53 as a cell death activator, it might be more achievable to identify easier targetable components upstream and downstream of p53. However, despite intensive research, it is still not clear which of the many p53 activators, target genes, and effector pathways are crucial for mediating tumor suppression. Furthermore, the key targets and pathways suppressing malignancy might be even context dependent; therefore, more research is needed to address which of the many p53 functions are crucial in specific tissues such as the colon. Due to the complexity of the p53 signaling network, a one-size-fits-all approach might not be suitable. Instead, the cellular and molecular context have to be taken into account when developing therapeutic approaches for cancer and CRC therapy. The challenges in the coming years of p53 research will be to deepen our understanding of p53 at the molecular and cellular levels and to translate this knowledge into clinical applications.

## Figures and Tables

**Table 1:** Overview of compounds targeting wild-type or mutant p53 in clinical trials in colorectal cancer or solid tumors (not specified). DN, dominant-negative; HLA, human leukocyte antigen; ICI, immune checkpoint inhibition; ID, identifier. IHC, immunohistochemistry; MM, multiple myeloma; TCR, T cell receptor.

Compounds Targeting p53 Wild-Type						
Compound	Combination Therapy	Disease	Phase	ClinicalTrials.Gov ID (accessed on 1 April 2021)	Inclusion Criteria Regarding p53 Status	(Possible) Mechanism of Action
AMG232	—	Solid tumor, MM	1	NCT01723020	p53 wild-type	MDM2 inhibition
APG-115	—	Solid tumor, lymphoma	1	NCT02935907	p53 mutation analysis	MDM2 inhibition
	Pembrolizumab	Solid tumor, melanoma	1, 2	NCT03611868	p53 wild-type	MDM2 inhibition + ICI
	Toripalimab	Solid tumor, liposarcoma	1, 2	NCT04785196	—^1^	MDM2 inhibition + ICI
BI 907828	—	Solid tumor	1	NCT03449381	p53 wild-type or unknown	MDM2 inhibition
	alone or plus BI 754,091 ± BI 754,111	Solid tumor	1	NCT03964233	p53 wild-type or unknown	MDM2 inhibition ± ICI
CGM097	—	Solid tumor	1	NCT01760525	p53 wild-type	MDM2 inhibition
HDM201	Alone or plus ancillary treatment	Solid or hematological tumors	1	NCT02143635	p53 wild-type	MDM2 inhibition
	PDR001^2^	CRC, other solid tumors	1	NCT02890069	—	MDM2 inhibition ± ICI
	Trametinib	CRC	1	NCT03714958	p53 wild-type	MDM2 + MEK inhibition
	Ribociclib	Solid tumor	2	NCT04116541	p53 wild-type	MDM2 + CDK4/6 inhibition
Idasanutlin (RG7388/ RO5503781)	—	Solid tumor	1	NCT02828930	—	MDM2 inhibition
	—	Solid tumor	1	NCT03362723	—	MDM2 inhibition
	—	Neoplasm (except leukemia)	1	NCT01462175	p53 mutation analysis	MDM2 inhibition
	Posaconazole	Solid tumor	1	NCT01901172	—	MDM2 inhibition + anti-fungal drug
	—^1^	Solid tumor, leukemia	1, 2	NCT04029688	—^1^	MDM2 inhibition
	Atezolizumab	CRC	1, 2	NCT03555149	—	MDM2 inhibition + ICI
	—	Solid tumor	2	NCT04589845	p53-wildtype	MDM2 inhibition
Milademetan (DS-3032b)	—	Solid tumor, lymphoma	1	NCT01877382	No known p53 mutation, p53 mutation analysis	MDM2 inhibition
MK-8242	—	Solid tumor	1	NCT01463696	p53 mutation analysis	MDM2 inhibition
RO5045337 (RG7112)	—	Solid tumor	1	NCT00559533	—	MDM2 inhibition
	—	Solid tumor	1	NCT01164033	—	MDM2 inhibition
	—	Neoplasm	1	NCT01677780	—	MDM2 inhibition
RO6839921 (RG7775)	—	Neoplasm	1	NCT02098967	—	MDM2 inhibition
SAR405838 (MI-77031)	—	Neoplasm	1	NCT01636479	—	MDM2 inhibition
	Pimasertib	Neoplasm	1	NCT01985191	—	MDM2 + MEK1/2 inhibition
ALRN-6924	—^3^	Pediatric cancer	1	NCT03654716	p53 wild-type	MDM2/4 inhibition
	Paclitaxel	Solid tumor	1	NCT03725436	p53 wild-type	MDM2/4 inhibition + chemotherapy
	—	Solid tumor, lymphoma	1, 2	NCT02264613	p53 wild-type	MDM2/4 inhibition
Ad-p53 (Gendicine)	—	CRC, other solid tumors	1	NCT01191684	>10% of p53-positive cells in IHC	Gene therapy to deliver p53 wild-type
	Pembrolizumab	CRC, other solid tumors	1	NCT02432963	p53 mutation or ≥10% of p53-positive cells in IHC	Gene therapy to deliver p53 wild-type + ICI
	Xeloda or Keytruda or Opdivo	Solid tumor	1, 2	NCT02842125	p53 wild-type or < 20% of p53-positive cells in IHC	Gene therapy to deliver p53 wild-type + chemotherapy or ICI
	Approved ICI	Solid tumor, lymphoma	2	NCT03544723	p53 wild-type or < 20% of p53-positive cells in IHC	Gene therapy to deliver p53 wild-type
**Compounds Targeting p53 Mutant**						
**Compound**	**Combination Therapy**	**Disease**	**Phase**	**ClinicalTrials.gov** **ID (accessed on 1 April 2021)**	**Inclusion Criteria regarding p53 Status**	**(Possible) Mechanism of Action**
PRIMA-1MET (APR-246)	Pembrolizumab	Solid tumor	1, 2	NCT04383938	p53 mutation	Restoration of wild-type functions + ICI
COTI-2	Alone or plus Cisplatin	CRC, other solid tumors	1	NCT02433626	—^2^	Restoration of wild-type functions + chemotherapy
Kevetrin (thioureidobutyronitrile)	—	Solid tumor	1	NCT01664000	—	Degradation of mutant p53 (activation of wild-type p53)
Atrovastatin	—	Ulcerative colitis	2	NCT04767984	DN missense p53 mutation	Degradation of mutant p53 + Inhibition of the melanovate pathway
Tanespimycin (17-AAG)	Irinotecan	Solid tumor	1	NCT00119236	—	Degradation of mutant p53 by HSP90 inhibition + chemotherapy (Investigation if response depends on p53 mutational status?)
Mutant p53 peptide pulsed dendritic cell vaccine		Colorectal cancer, other solid tumors	2	NCT00019084	p53 mutation	Mutant p53 vaccine
ALT-801	—	Neoplasm	1	NCT00496860	HLA-A2.1/p53 positive	IL-2 fused to TCR recognizing p53

^1^ Patients with solid tumors. ^2^ Patients with CRC. ^3^ All enrolled patients except p53 wild-type acute leukemia.

## Supplementary Materials

The following are available online at https://www.mdpi.com/article/10.3390/cancers13092125/s1, Figure S1: Prevalence of genetic alterations in p53 and upstream p53 regulators in COAD.

## Abbreviations

5-FU	5-Fluorouracil
aa	amino acid
ASPP	apoptosis-stimulating of p53 protein
CSC	cancer stem cell
CIMP	CpG island methylator phenotype
CIN	chromosomal instability
CMS	Consensus Molecular Subtype
COAD	colorectal adenocarcinoma
CRC	colorectal cancer
CTD	C-terminal domain
DBD	DNA-binding domain
DN	dominant-negative
DNE	dominant-negative effect
GOF	gain-of-function
HLA	human leukocyte antigen
HZF	hematopoietic zinc finger
IARC	International Agency for Research on Cancer
ICI	immune checkpoint inhibitor
IHC	immunohistochemistry
lncRNA	long non-coding RNA
LOF	loss-of-function
MHC I	major histocompatibility complex class I
miRNA	microRNA
MM	multiple myeloma
MSI	microsatellite instability
ncRNA	non-coding RNA
OD	oligomerization domain
PKC	protein kinase C
PLK1	polo-like kinase 1
PRD	proline-rich domain
PTM	post-translational modification
RE	response element
ROS	reactive oxygen species
TAD	Transactivation domain
TCR	T-cell receptor
TF	transcription factor
