# Supplementary material for: The Role of p53 Signaling in Colorectal Cancer
Source: Cancers (Basel). 2021 Apr 28;13(9):2125. doi: 10.3390/cancers13092125 (PMC8125348; doi:10.3390/cancers13092125)
Supplement: Supplementary file 1 [file cancers-13-02125-s001.zip › cancers-1188407Figure S1.pdf]

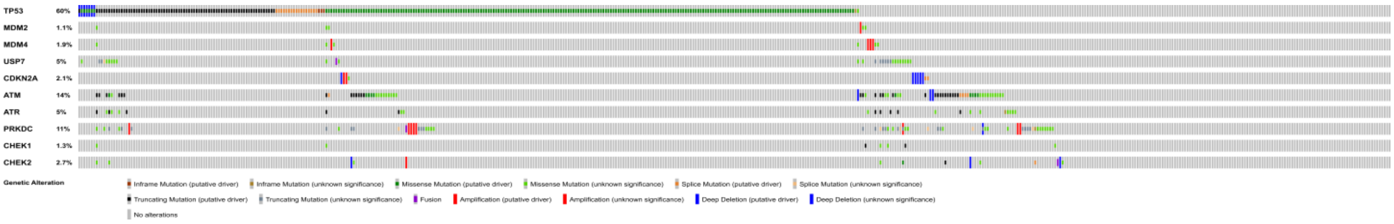

Figure S1: Prevalence of genetic alterations in p53 and upstream p53 regulators in COAD. The Oncoprint displays the genomic alterations (legend) in p53 and upstream p53 regulators (rows) across 526 COAD samples (columns; TCGA, PanCancer COAD dataset). The data was accessed via the cBioPortal webservice (https://www.cbioportal.org/) [180,181].
